# Detection of Rat Pain-Related Grooming Behaviors Using Multistream Recurrent Convolutional Networks on Day-Long Video Recordings

**DOI:** 10.3390/bioengineering11121180

**Published:** 2024-11-21

**Authors:** Chien-Cheng Lee, Ping-Wing Lui, Wei-Wei Gao, Zhongjian Gao

**Affiliations:** 1 Department of Electrical Engineering, Yuan Ze University, Taoyuan 320, Taiwan; 2 St. Paul’s Hospital, Taoyuan 330, Taiwan; 3 School of Mechanical and Electrical Engineering, Sanming University, Sanming 365004, China

**Keywords:** action recognition, convolutional neural network, multistream recurrent network, pain studies, rat behaviors

## Abstract

In experimental pain studies involving animals, subjective pain reports are not feasible. Current methods for detecting pain-related behaviors rely on human observation, which is time-consuming and labor-intensive, particularly for lengthy video recordings. Automating the quantification of these behaviors poses substantial challenges. In this study, we developed and evaluated a deep learning, multistream algorithm to detect pain-related grooming behaviors in rats. Pain-related grooming behaviors were induced by injecting small amounts of pain-inducing chemicals into the rats’ hind limbs. Day-long video recordings were then analyzed with our algorithm, which initially filtered out non-grooming segments. The remaining segments, referred to as likely grooming clips, were used for model training and testing. Our model, a multistream recurrent convolutional network, learned to differentiate grooming from non-grooming behaviors within these clips through deep learning. The average validation accuracy across three evaluation methods was 88.5%. We further analyzed grooming statistics by comparing the duration of grooming episodes between experimental and control groups. Results demonstrated statistically significant changes in grooming behavior consistent with pain expression.

## 1. Introduction

Pain is a fundamental symptom of many diseases, including cancer, yet its physiological mechanisms remain largely unclear. While human studies have provided some insights, most major advances in understanding the pathophysiology of pain have come from animal research, where deeper questions can be explored and controlled experimental manipulations can be conducted. However, unlike humans, animals cannot provide subjective pain reports, making the objective assessment of pain in animals a significant challenge. Our research specifically aims to address this challenge.

In experimental pain studies involving animals, subjective reporting is not feasible. Therefore, pain-related reflexes or behaviors are typically used as indicators of pain. For rodents, typical pain-related behaviors include repetitive licking or grooming of body parts affected by pain stimuli, as well as lifting or flinching of the hind paw after injection of a pain inducer (e.g., 5% formalin) into the dorsal or plantar surface of the paw [1]. Such nociceptive behaviors consist of three temporal phases: phase 1 (~5 to 10 min) followed by a quiescent period (~5 min), phase 2 (~30 to 40 min), and phase 3 (lasting one or more days) of hypersensitivity [2]. While behavioral changes are well established in phases 1 and 2, phase 3 behaviors remain poorly analyzed, likely due to the lengthy datasets involved. The inflammatory response to a local injection of carrageenan into a rat paw was introduced by Winter et al. [3], and it has become a widely used model of acute inflammation, a process involving mast cell mediators, prostaglandins, kinins, neuropeptides, and nitric oxide [4,5,6].

Currently, detecting grooming behavior relies on subjective human judgment, often requiring time-consuming and labor-intensive viewing of lengthy video recordings, especially in cases of long-lasting chronic pain. Consequently, only selected portions of the recordings are typically analyzed, rather than the entire video. This selective analysis raises concerns about the validity of extrapolating findings from shorter time frames (e.g., pain phases 1 and 2) to longer periods (e.g., phase 3). Automating the quantification of such behavior presents a significant challenge. To address this, we developed and tested a computer-based algorithm to detect pain-related grooming behaviors by analyzing ~24 h video recordings, covering all three pain phases.

Several methods have been proposed to detect rat behaviors. For example, Doppler radar signals can be used as input to a multilayer feedforward neural network to discriminate rat behaviors like walking, grooming, and stillness [7]. Other methods include using a wireless tri-axial accelerometer worn by the rat, where 3D acceleration data are analyzed to recognize natural behaviors in a cage. Neural network-based pattern recognition algorithms have successfully identified eating, grooming, and standing behaviors [8]. Another experimental approach automatically measures rats’ position, grooming frequency, and rearing activity through vibration-based grooming sensors, infrared LED-based rearing sensors, and active tracking cameras [9]. Currently, the most widely used approach for rat behavior recognition combines cameras with computer vision and machine learning algorithms [10,11,12]. Motion sensing devices, such as 3D depth cameras (e.g., Microsoft Kinect), provide a more powerful alternative to traditional RGB cameras [13,14].

Most experimental studies focus on algorithm development, with test data that are typically short in duration (<4 h). Consequently, these algorithms are not designed for analyzing long datasets, as required in experimental pain studies (e.g., 24 h to capture the daily rhythm of rodents).

In recent years, the increasing use of deep learning and convolutional neural networks (ConvNets) in image analysis has significantly advanced visual recognition for behavioral studies. Compared to traditional machine learning algorithms, ConvNet models, which are based on “self-learning”, can substantially improve the accuracy of behavior recognition. Pham et al. [15] provides a thorough review of deep learning approaches in video-based action recognition, covering key models and datasets that have significantly advanced the field. It discusses convolutional and recurrent networks commonly applied to human motion and behavior detection in videos. To capture both spatial and temporal features, various approaches have been applied to extend ConvNet connectivity into the time domain, thereby leveraging local spatial–temporal information [16]. Additionally, several algorithms have been proposed to enhance performance, such as stacking contiguous video frames and fusing different spatial–temporal information (e.g., single frame, early fusion, slow fusion, and late fusion). Donahue et al. proposed a visual behavior recognition method based on Long-term Recurrent Convolutional Networks (LRCNs) [17]. This method combines the rapid processing capabilities of ConvNets to capture both image and optical flow features with the sequence-learning ability of long short-term memory (LSTM) networks to extract temporal information in videos for behavior recognition. In another study, Simonyan and Zisserman introduced a two-stream ConvNet method [18] that significantly improves recognition accuracy by using two ConvNet inputs: (1) RGB spatial images and (2) frame optical flow patterns.

The two-stream architecture is fundamental to many current models for behavior recognition, with a variety of recognition models derived from this framework. For example, one two-stream ConvNet model extracts spatial and temporal features, which are then input to an LSTM model, and the results are fused for the final output [19]. Different fusion methods for spatial and temporal features have also been proposed [20]. Hu et al. [21] provide a comprehensive overview of the field, highlighting different neural network architectures, including two-stream convolutional networks and LSTM networks, used in behavior recognition. A temporal segment network (TSN) model was introduced as well [22]. In this approach, a video is divided into several time segments, with fewer images analyzed to reduce computational load. A two-stream ConvNet then extracts features from these image segments, which are subsequently fused to generate the output. An attention mechanism was later added to these models to enhance performance [23,24]. More recently, Ma et al. proposed a TS-LSTM model [25] in which spatial and temporal features are extracted with a two-stream ConvNet, fused, and then divided into segments. After batch normalization and temporal pooling of individual segments, the processed data are fed into an LSTM for the final classification.

On the other hand, most image and video classification or action recognition methods rely on labeled datasets, which can be time-consuming and labor-intensive to create, especially for large datasets. Few-shot learning, however, seeks to mimic the human ability to learn from just a few examples, unlike traditional supervised learning. For example, Li et al. [26] proposed a knowledge-guided semantic transfer network (KSTNet) for few-shot image recognition, integrating vision inference, knowledge transfer, and classifier learning into a unified framework for optimal compatibility. Tang et al. [27] introduced mechanisms to learn generalized and discriminative feature embeddings, enhancing classifier robustness against prediction errors in meta-learning. More recently, advanced research [28,29,30,31] has focused on few-shot fine-grained recognition, which aims to identify novel, fine-grained categories with limited samples.

Most visual recognition studies of behavior use the standard UCF101 dataset [32], for both training and testing models. The UCF101 dataset consists of color video clips of 101 human actions, available online as web videos. These actions are categorized into five types—human–object interaction, body motion, human–human interaction, playing musical instruments and sports—with an average clip length of around 7 s. This dataset differs significantly from our recorded videos of rat behaviors. First, due to the experimental requirements, our rat video recordings were much longer (~24 h), with the rat being motionless or actively moving for most of the time. Second, rat grooming behavior involves smaller amplitude motions compared to human behaviors. Third, our videos were collected in monochrome (instead of color) and had lower spatial resolutions. Because of these differences, methods proposed in the literature cannot be directly applied to our pain study.

We proposed a two-stage activity detection method specifically for identifying rat grooming behavior. In the first stage, a day-long video recording was divided into three types of short clips based on a simple index derived from frame-to-frame differences: motionless, drastic movement, and small movements. The small movement clips were labeled as “likely grooming clips”, as they likely contained grooming activity. A human review of these “likely grooming clips” further classified them as either “confirmed grooming clips” or “confirmed non-grooming clips”. In the second stage, a grooming activity detection algorithm was trained and tested on these confirmed clips in either a dataset-dependent or dataset-independent manner. Because small movement clips were fewer in number than the other two types (motionless and drastic movement), the processing time for analyzing day-long video recordings was greatly reduced.

Our working hypothesis is twofold: (1) The proposed two-stage activity detection method can detect grooming episodes with a satisfactory level of accuracy; and (2) Pain-related activity is indicated by an increased frequency of episodes within a specific range of grooming duration, likely occurring when the animal is licking body parts affected by pain.

## 2. Materials and Methods

Our study protocol was approved by the Institutional review board of the Veterans General Hospital (approval code: LA-1071539, 11 January 2018).

### 2.1. Data Acquisition

#### 2.1.1. Animals

**Figure 1:**
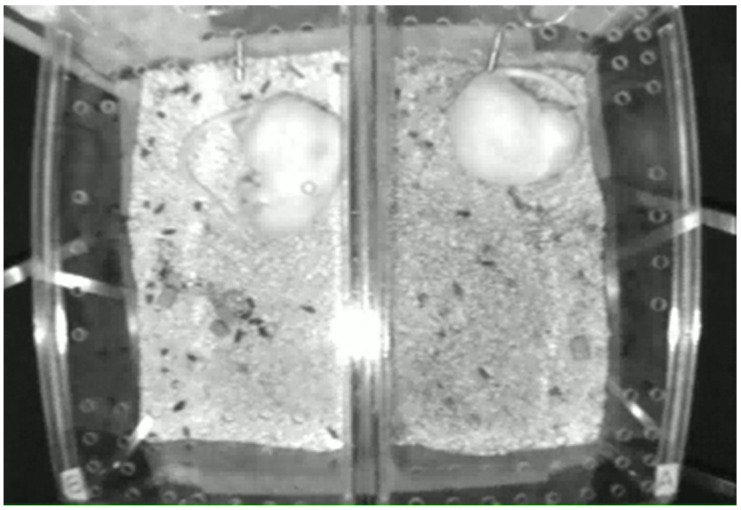
Top view of a video image showing two rats housed in separate rearing cages (**left**: experimental; **right**: control).

In our study, we used videos from previous animal experiments related to pain, where pain-related grooming in rats was induced by injecting small amounts of pain-inducing chemicals into their hind limbs. Male adult rats (Sprague Dawley, body weight 250–300 gm, *n* = 12), were randomly assigned to 6 groups, each of which was video-recorded to create a dataset. Each group consisted of two rats, randomly assigned as either experimental or control. The rats were placed in top-open cages (Figure 1), positioned side-by-side in a quiet laboratory room. The transparent cages were equipped with water and food supplies, and a video camera was mounted above each cage. Room temperature and humidity were controlled (~25 °C, 40–75%) under a 12 h light/dark cycle. Datasets for each group consisted of three daily video recording sessions across three consecutive days: (1) the first two days as the ‘pre-drug’ condition; and (2) the third day as the ‘post-drug’ condition.

#### 2.1.2. Pain Induction

Two pain inducers were used in the study—5% formalin (a short-term, strong acute pain inducer) and 1% or 3% carrageenan (a long-acting chronic pain inducer)—following established protocols for experimental pain [33,34]. Carrageenan is more effective for inducing pain of longer duration, while formalin is more effective for inducing pain of shorter duration [33,34]. On the third day, the dorsal surface of the hind paw on one side of the experimental rat was subcutaneously injected with 0.2 mL of the respective pain inducer (administered via a 27-gauge syringe). Control rats received similar injections of 0.9% physiological saline. To minimize animal suffering, the injection was performed under inhalation anesthesia using a short-acting drug (isoflurane). Rats were placed in the induction chamber with oxygen flow set at 1.0 L/min; isoflurane was administered at 4% for induction and 2% for maintenance. After the injections, rats were allowed to recover from anesthesia before being returned to their original cages. If any signs of distress were observed (e.g., difficulty breathing, cramping), the animals were removed from their cages and physically examined, including the injection sites, to determine whether the experiment could continue. If necessary, euthanasia would be performed; however, none of the rats were removed from the experiment prematurely.

#### 2.1.3. Video Recordings

Monochrome video images of the experimental animals were captured using a digital camera mounted above the open-top cages, with a frame rate of 1 frame per second (320 × 240 pixels). Figure 1 shows an example of the captured frame images. Each dataset consisted of three day-long video recordings (two days of pre-drug condition and one day of post-drug condition), during which two rats (one experimental and one control) were placed side-by-side in separate cages, with both rats imaged from above. Between daily sessions, video recordings were paused for approximately 20 to 90 min to allow for cage cleaning, bedding replacement, replenishment of food and water, and checking and cleaning of the injection sites. Table 1 provides details of the injections for the various animal groups and the lengths of their recorded videos. Our choice of relatively low spatial resolution (320 × 240 pixels) and longer sampling intervals (1 frame/s) was made to enable the extension of our method to datasets obtained from large-scale experiments, where multiple animals are recorded simultaneously.

### 2.2. Two-Stage Grooming Activity Detection Scheme

**Figure 2:**
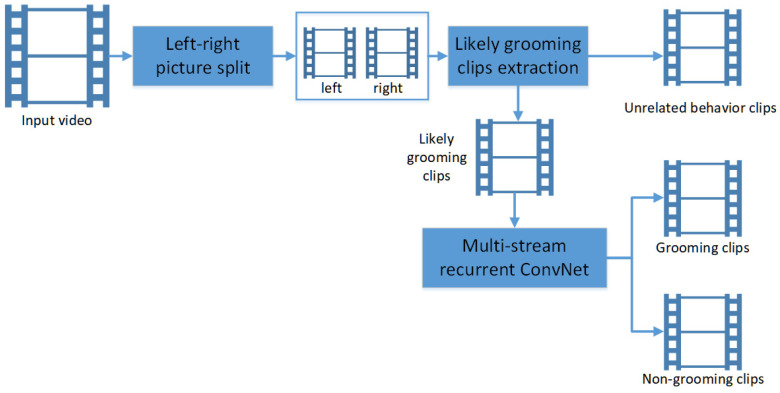
Overview of the two-stage grooming activity detection scheme.

The method is a two-stage grooming activity detection scheme developed for analyzing day-long video recordings. It is designed to be faster and more memory-efficient and to offer improved performance in recognizing grooming behavior in rats compared to existing methods. An overview of the scheme is shown in Figure 2.

In the first stage of processing, a time series reflecting movement is generated to identify likely grooming clips. A simple index, the consecutive frame difference, is calculated to exclude frames unrelated to grooming (details are provided later). This step significantly reduces data processing time. After the first stage, only the likely grooming clips remain. These clips are then subjectively classified by an experimenter into one of two categories: confirmed grooming clips or confirmed non-grooming clips.

In the second stage of processing, three data-splitting approaches are used to prepare training and validation data from the human-confirmed clips (grooming and non-grooming) for training and evaluating the multistream recurrent ConvNet to recognize grooming activity. The following are the processing steps described in more detail.

#### 2.2.1. Extraction of Likely Grooming Clips

The adjacent frame difference calculates the difference between two consecutive frames in a video sequence. In this method, the calculation is first performed to generate time series (signals reflecting cross-frame motion) for the left or right image, respectively. Given a frame *I* size of W×H, the metric of frame difference between two consecutive frames, $d_{p}\left(t\right)$ is defined as

(1) $$d_{p}\left(t\right)=\frac{1}{W\times H}\sum_{i=1}^{W}\sum_{j=1}^{H}sign\left(\left|I_{t}\left(i,j\right)-I_{t+1}\left(i,j\right)\right|-\alpha \right),$$

where $p\in \left\{left,\;right\right\}$, $I_{t}\left(i,j\right)$ denotes the intensity of a pixel at location $\left(i,j\right)$ in the *t*-th frame and 1≤t<T covers the entire length of the recorded video that contains *T* frames. *sign* is the sign function. The parameter α is a minimum difference tolerance, which is taken to exclude from processing those frames with pixel gray-level differences <α generated inevitably by camera sensor noise.

The overlapping moving window technique is commonly used in time-series analysis to analyze data in sequential segments while preserving continuity. This method helps identify subtle and gradual changes in behavior. To exclude frames containing multiple abrupt or sudden movements, we employ a relatively long overlapping moving window (10 frames, as described later) to calculate the moving average $D_{p}\left(s\right)$ of the *s*-th window:

(2) $$D_{p}\left(s\right)=\frac{1}{w}\sum \left[d_{p}\left(\left(s-1\right)\times k+1\right),d_{p}\left(\left(s-1\right)\times k+2\right),\ldots ,d_{p}\left(\left(s-1\right)\times k+w\right)\right]$$

where *k* is the step-size, and *w* is the window size of the 50% overlapping moving window. Two pre-defined thresholds are empirically set to extract the likely grooming episodes: the minimal frame difference ($\theta_{min}$) and the maximal frame difference ($\theta_{max}$). If the moving average $D_{p}\left(s\right)$ is smaller than the minimal frame difference, the rat is presumed to be motionless or at rest. Conversely, if $D_{p}\left(s\right)$ exceeds the maximal frame difference, the rat is assumed to be engaged in fast movements with large excursions.

A likely grooming indicator $g_{p}\left(s\right)$ is defined to indicate whether a frame is likely related to grooming (with a value of “1”) or unrelated to grooming (with a value of “0”). This is determined according to the following equation:

(3) $$g_{p}\left(s\right)=\left\{\begin{array}{c} 1\;\theta_{min}<D_{p}\left(s\right)<\theta_{max}\\ 0\;otherwise \end{array}\right..$$

Rats housed in a cage tend to pause or make quick jerking movements lasting less than 5 s during grooming or non-grooming episodes. These instances are considered as brief interruptions, or “contaminations”, within otherwise continuous motion or motionless episodes. To avoid breaking up these episodes due to such brief interruptions, each 5-s contaminated segment is compared to its neighboring 5-s segments (already labeled as likely grooming or likely non-grooming). A 3-point median filter, covering three 5-s segments, is applied twice to $g_{p}\left(s\right)$, resulting in a smoothed indicator ${\hat{g}}_{p}(s)$. In the final output, likely grooming clips are represented by continuous sequences in ${\hat{g}}_{p}(s)$ with values “1” (indicating grooming) rather than being interrupted by isolated “0” s. The resulting set of likely grooming clips is defined as follows:

(4) $$E_{p}=\left\{e_{pk}|k=1,2,\cdots ,N_{p}\right\},$$

where $N_{p}$ is the total number of the likely grooming clips and $p\in \left\{left,\;right\right\}$. Similarly, the likely non-grooming segments form a continuous series ${\hat{g}}_{p}(s)$ with all values “0”.

**Figure 3:**
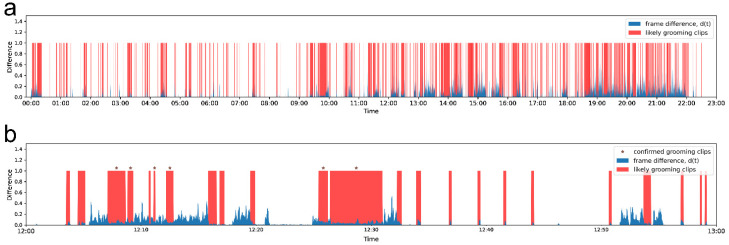
An example of the time profile series. (**a**) Day-range data; (**b**) Hour-long data.

**Figure 4:**
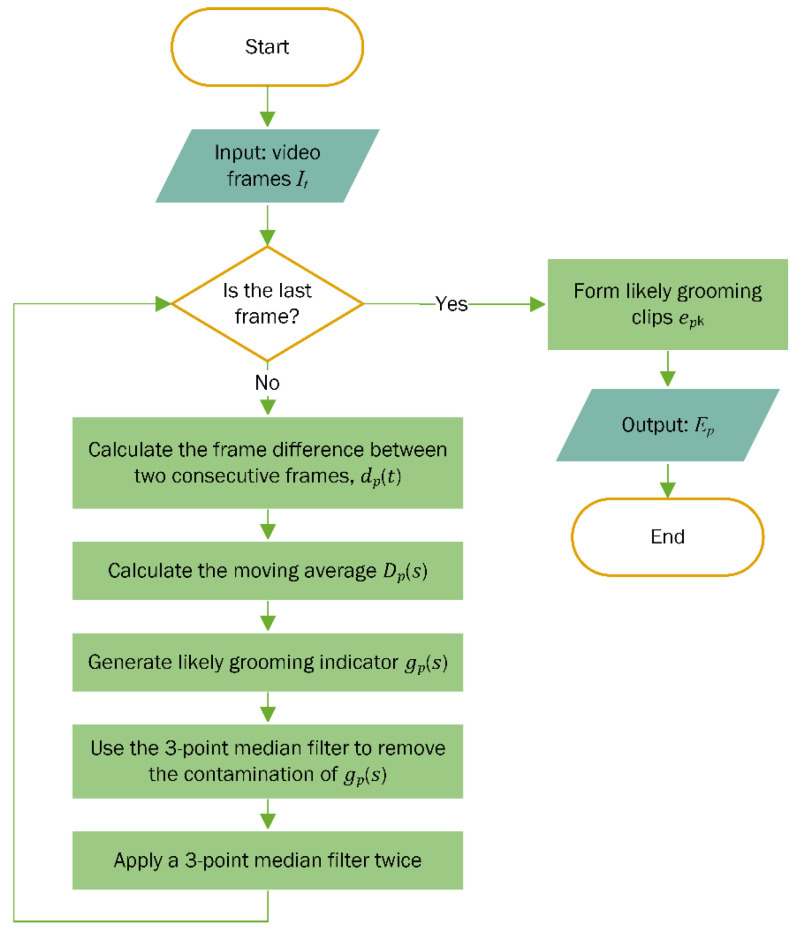
Flowchart of extracting likely grooming clips.

To exclude 5-s non-grooming segments that may appear twice within a longer likely grooming segment, a 3-point median filter is applied twice. This filtering process ensures that the shortest “likely grooming clip” is at least 10 frames long. Additionally, any isolated likely grooming clips of only 5 s are either excluded or interpolated with the surrounding likely non-grooming background. Figure 3 presents an example of the time-series profile for frame difference $d_{p}\left(s\right)$ combined with likely grooming clips $E_{p}$. In this figure, blue areas represent individual frame difference values, while red areas indicate likely grooming clips $e_{pk}$. Thresholds are set at $\theta_{min}$ = 0.009 and $\theta_{max}$ = 0.08. Asterisks mark confirmed grooming clips containing grooming activity as verified by the experimenter. The flowchart for extracting likely grooming clips is summarized in Figure 4.

#### 2.2.2. Architecture of the Multistream Recurrent ConvNet

**Figure 5:**
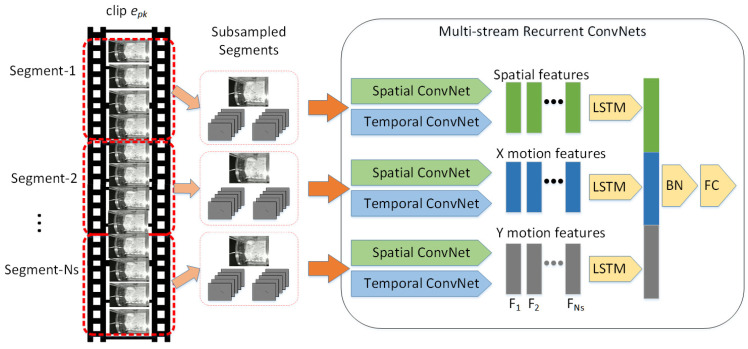
Architecture of the proposed MSRC for grooming behavior recognition.

The architecture of our multistream recurrent ConvNets (MSRCs) for recognizing grooming behavior is illustrated in Figure 5. The MSRC model employs spatial ConvNets to extract spatial features and temporal ConvNets to capture motion features. Initially, the input video clip $e_{pk}$ identified as a likely grooming clip is divided into $N_{s}$ shorter segments of equal length. Since our rat recordings are in monochrome, three frames are randomly selected from each segment to construct a 3-channel image, $A_{n}$, where *n* = 1, 2, …, $N_{s}$. Additionally, for each segment, a sequence of $N_{f}$ frames is selected, starting at a random offset, to generate stacked optical flow fields in the *x*- and *y*-directions, denoted as $O_{n}^{x}$ and $O_{n}^{y}$, respectively.

Through this process, the 3-channel images $A_{n}$ encapsulate the spatial information, while the stacked optical flow fields $O_{n}^{x}$ and $O_{n}^{y}$ provide the motion information. Subsequently, the 3-channel images are fed into spatial ConvNets to extract spatial features $F_{n}^{A}$. Similarly, the stacked optical flow fields are input into temporal ConvNets to derive motion features $F_{n}^{x}$ and $F_{n}^{y}$. These feature representations are defined as follows:

(5) $$F_{n}^{A}={ConvNet}_{s}\left(A_{n}\right),$$

(6) $$F_{n}^{x}={ConvNet}_{t}\left(O_{n}^{x}\right),$$

(7) $$F_{n}^{y}={ConvNet}_{t}\left(O_{n}^{y}\right),$$

where ${ConvNet}_{s}$ represents the spatial ConvNets responsible for extracting spatial features, and ${ConvNet}_{t}$ represents the temporal ConvNets that extract motion-related features.

To leverage the temporal sequence and minimize variations, we use a recurrent neural network (RNN), specifically an *LSTM*, to capture temporal dynamics by mapping the feature inputs to hidden states. Spatial and motion feature vectors $F_{n}^{A}$, $F_{n}^{x}$, and $F_{n}^{y}$ with time step $N_{s}$ serve as inputs to the *LSTM*. The *LSTM* encodes these inputs, and the final hidden state represents the temporal dynamics of the $N_{s}$ segments. The three final hidden state vectors are then concatenated into a single vector and passed through batch normalization (*BN*) and fully connected (*FC*) layers to produce the final prediction. The network output *Y* is computed as follows.

(8) $$Y\left(F^{A},F^{x},F^{y}\right)=FC\left(BN\left(concat\left(LSTM\left(F^{A}\right),LSTM\left(F^{x}\right),LSTM\left(F^{y}\right)\right)\right)\right).$$

The MSRC model provides a general and adaptable framework for video-level behavioral recognition. ConvNets serve as the backbone of this model and can be replaced with several advanced architectures, such as VGGNet [35], GoogleNet [36], ResNet [37], DenseNet [38], and ResNeXt [39]. For our study, we selected ResNet-50 as the ConvNet architecture due to its balanced trade-off between accuracy and computational efficiency, which is crucial for processing extensive video data.

Most ConvNets offer pre-trained models on large datasets like ImageNet, but these models have not encountered rat video data, which poses a domain difference. Transfer learning enables efficient and accurate model training for new, specialized datasets with limited labeled data. In our approach, we fine-tuned the pre-trained ConvNet by unfreezing and training the last convolutional block, retaining the broad, generic features learned from ImageNet while allowing the model to adapt to the domain-specific nuances of rat behavior. This strategy both accelerated the training process and improved the accuracy of grooming behavior recognition in the rat videos.

In this study, we set the clip segments $N_{s}$ to 3 and the length of stacked optical flow fields $N_{f}$ to 10 to capture both short and intermediate-term motion, which is critical for accurately recognizing grooming behaviors. These values were chosen based on prior studies indicating that a moderate number of segments and flow fields helps balance computational efficiency with sufficient temporal detail. The Adam optimizer with learning rate 0.0005 is used to train the network parameters. This learning rate was determined based on preliminary testing, where it provided an optimal balance between convergence speed and generalization. We selected a batch size of 25 to optimize GPU memory usage while maintaining stable gradient estimates during training. The number of epochs was set to 30 based on early stopping criteria, which allowed the model to reach satisfactory performance without overfitting.

**Figure 6:**
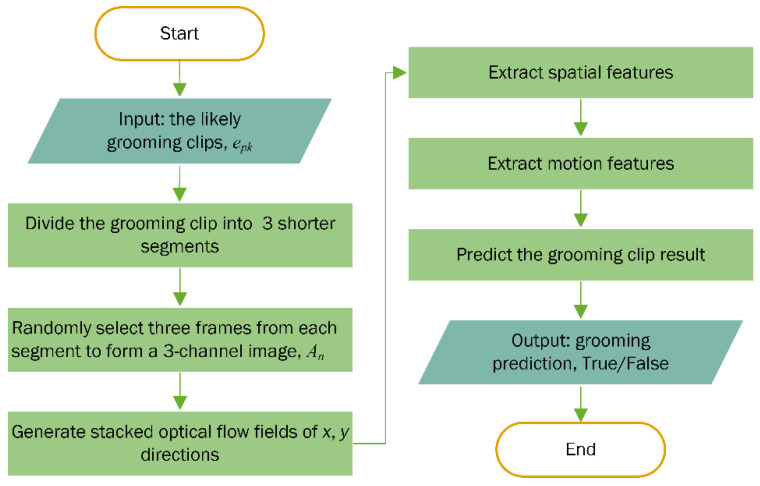
Flowchart of MSRC, grooming behavior prediction.

The spatial ConvNet weights in our model were initialized with pre-trained ImageNet weights, leveraging transfer learning to enhance feature extraction from the new dataset. For temporal ConvNets, we averaged the pre-trained model’s RGB channel weights, providing an effective initialization for handling our monochrome rat video data. The LSTM layer, which captures temporal dynamics, had 64 hidden units, used the tanh activation function, and operated with a time step of 3. We implemented this MSRC model using the PyTorch 2.3.0 framework and performed training and evaluation on a system equipped with an Intel(R) Xeon Silver 4110 CPU (Intel Corporation, Santa Clara, CA, USA) and an NVIDIA Tesla V100-32 GB GPU (NVIDIA Corporation, Santa Clara, CA, USA). A summary of the MSRC model’s flow is presented in Figure 6.

#### 2.2.3. Generation of Grooming Duration Histograms

**Figure 7:**
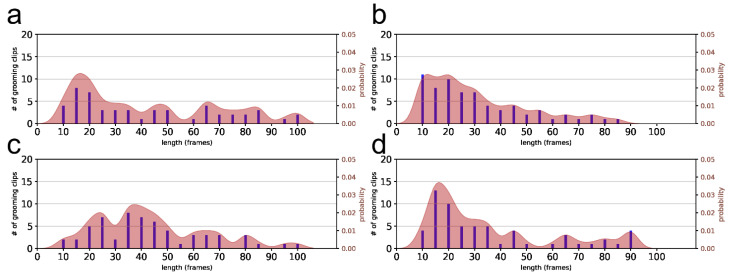
Grooming duration histograms. (**a**) Pre-drug experimental, (**b**) pre-drug control, (**c**) post-drug experimental, and (**d**) post-drug control. The bars represent the histograms, while the curves depict the estimated PDF profiles. Note different y-scales in each panel.

To examine the grooming clip duration distribution, we categorized the clips into pre-drug and post-drug conditions. For a clearer comparison, pre-drug grooming clips were divided by two to match the single-day post-drug observations, as summarized in Table 2. Figure 7 illustrates an example histogram of grooming clip durations for the Frmln-A dataset, focusing on clips lasting ≤100 s. Despite the extensive recording periods, the grooming observations were sparse in some duration bins, likely due to the overlapping window approach in our grooming extraction method.

To refine the histogram data, we applied kernel density estimation (KDE) using a Gaussian kernel with a bandwidth of 3, which produced a probability density function (PDF) that smooths the distribution for more robust statistical interpretation. This KDE-derived PDF for dataset Frmln-A is also shown in Figure 7, and additional PDFs for other datasets are provided in Appendix A.

## 3. Experiments and Results

In this section, we begin by evaluating the performance of our two-stage grooming behavior detection scheme across all six datasets to assess its effectiveness in recognizing grooming activities from day-long video recordings. Subsequently, we analyze grooming patterns for individual rats, comparing pre- and post-drug conditions to identify changes in behavior. We further extend this comparison to distinguish grooming behaviors between experimental rats (exposed to pain-inducing agents) and control rats. Finally, based on these results, we propose a response metric that reflects pain-related changes in grooming with statistical significance.

### 3.1. Evaluation of Two-Stage Grooming Behavior Detection

After extracting the likely grooming clips, we filtered out frames that were unrelated to grooming by analyzing both halves of each frame. The remaining likely grooming clips were then collected and processed. The statistics of these clips across the six datasets are summarized in Table 3. A total of 18,610 likely grooming clips were segmented from the datasets. On average, approximately 22% of the frames were retained, as these frames likely contained grooming behaviors. The maximum clip duration observed was 930 frames. Among these clips, 1031 were longer than 100 frames (5.54%). To simplify the analysis, these longer clips were excluded from the histograms depicting clips with durations of 100 frames or less. This focus on shorter grooming episodes ensures a clearer representation of the grooming patterns typically observed in the study.

The proposed MSRC model was used to classify the likely grooming clips as either true grooming or false grooming. To train and evaluate the MSRC model, each likely grooming clip was reviewed by the experimenter and labeled as either a confirmed grooming clip or a confirmed non-grooming clip. This classification took into account the video images preceding and/or following each given clip, ensuring a more accurate categorization.

**Figure 8:**
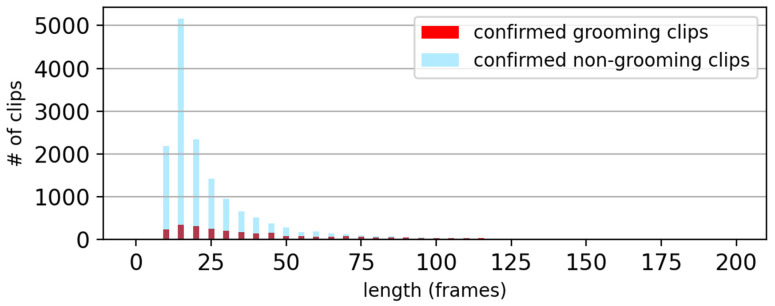
Duration histogram showing distributions of confirmed grooming clips and confirmed non-grooming clips of all six datasets.

To assess the performance of the model in differentiating confirmed grooming and confirmed non-grooming clips, we examined the duration distribution of these two types of clips. All the data from the three days of video recording (including both experimental and control groups) were pooled together. Duration histograms were then constructed for both confirmed grooming clips and confirmed non-grooming clips, as shown in Figure 8. These histograms provide insight into the characteristics of grooming behavior in terms of clip duration and help in understanding the overall grooming patterns in the study.

To evaluate the performance of the proposed model, we applied three different data-splitting approaches for training and validation: dataset-dependent, dataset-independent, and group-independent. The key difference between these approaches lies in which data are used for training and which data are used for validation. These three approaches are explained in more detail as follows:

1. Dataset-Dependent Training: In this approach, the data used for training are taken from part of each dataset, while the remaining portion of the same dataset is used for validation.
2. Dataset-Independent Training: No data from the validation dataset are used in the training process. This means that the training data and validation data are completely independent, and the model is tested on data from a completely different set than what it was trained on.
3. Group-Independent Training: Similarly to dataset-independent training, this approach ensures that the training and validation data are from different groups. The key difference is that grouping is based on the type of pain inducer or dose used.

These three splitting approaches provide a comprehensive evaluation framework for assessing the model’s performance under different validation conditions.

#### 3.1.1. Dataset-Dependent Evaluation

In the training and evaluation process, the confirmed grooming and confirmed non-grooming clips for each dataset were randomly split into a training set and a validation set at a ratio of 4:1, ensuring that both sets contained similar proportions of grooming and non-grooming clips. After splitting each dataset, all the training sets from the six datasets were merged to create a unified training dataset. Similarly, the validation sets from all the datasets were merged to create a validation dataset. These datasets were shuffled before training to avoid any potential biases in the order of the clips. Table 4 summarizes the number of clips in both the training and validation datasets (14,885 clips for training and 3725 for validation). These clips were used to train and evaluate the performance of the MSRC model.

We compared our MSRC model for grooming behavior recognition with other existing methods, and the results are summarized in Table 5. Our approach was benchmarked against models such as TSN and TS-LSTM. The training time for our model was approximately 1.5 h, with a training accuracy of 0.911 and a validation accuracy of 0.900, outperforming the other methods. Our model is inspired by the TSN model, which uses only BN-Inception as its ConvNet backbone. While TSN divides the input video clip into several segments, it does not include an LSTM for processing temporal data and relies on voting for the final prediction across the entire video. In contrast, the TS-LSTM model uses ResNet-101 as its ConvNet backbone, splitting the feature matrix after the ConvNet into several temporal segments. These segments are then pooled using max-pooling layers, and the outputs are sequentially fed into the LSTM layer. Our approach strikes a balance between efficient temporal segmentation (like TSN) and sophisticated temporal processing (like TS-LSTM), which likely enables our model to outperform both in terms of capturing spatial and temporal features effectively.

#### 3.1.2. Dataset-Independent Evaluation

In each training session, a single dataset is systematically reserved for validation, while the remaining datasets are used for training. The training data are shuffled before the training process begins. Evaluation is performed for each of the different combinations, which requires six times the computational load compared to the dataset-dependent approach. The evaluation results are shown in Table 6. The average training accuracy is 0.909, and the average validation accuracy is 0.882.

#### 3.1.3. Group-Independent Evaluation

The six datasets are grouped into three categories based on the pain inducers used: (1) 3% carrageenan group, (2) 1% carrageenan group, and (3) 5% formalin group. In each training session, one of these three groups is systematically used as the validation data, while the remaining two groups are used for training. The training data are shuffled before the training process begins. Evaluation is performed for each combination, and the results are shown in Table 7. The average training accuracy is 0.909, and the average validation accuracy is 0.879.

**Figure 9:**
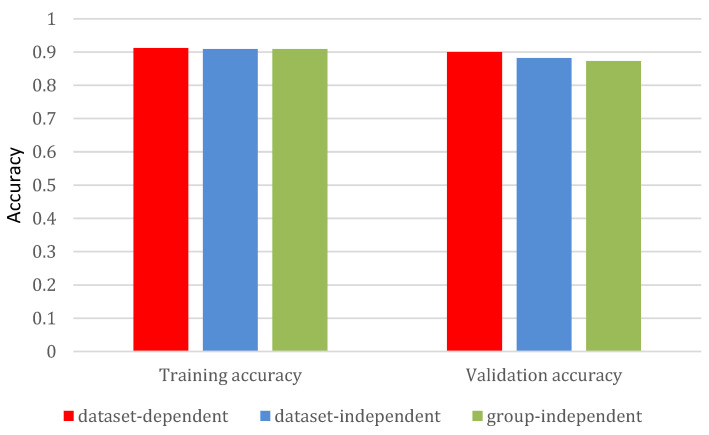
Graphic representation of results from the three evaluation approaches.

Figure 9 compares the three approaches based on average accuracy. The training accuracies are generally comparable, ranging from 0.873 to 0.900. As expected, the dataset-dependent evaluation achieves the highest validation accuracy (0.900), followed by the dataset-independent approach (0.882), while the group-independent evaluation performs the least well (0.873).

All three evaluations consistently show a small gap in accuracy (<3%) between training and validation, indicating a good generalization ability of the model to unseen data. This generalization is crucial for future animal experiments.

In the first stage of our video analysis, approximately 22% of frames are retained as likely grooming clips. In the second stage, the MSRC model performs well in identifying grooming behavior, with only around 10% misclassification on grooming clips. The proposed two-stage grooming activity detection scheme thus significantly reduces the need for human effort in identifying grooming clips from lengthy video recordings.

Grooming behaviors are successfully detected as individual clips using our model. These machine-detected grooming clips are then cross-checked manually. The human-confirmed grooming behaviors typically involve repetitive or sporadic motions such as licking, scratching, or rubbing body parts like the head, vibrissae, neck, limbs, abdomen, or tail (examples can be found in Supplementary Video S1). These movement patterns align with findings in the rat grooming literature [40,41]. Falsely identified grooming clips, however, are often due to poor visualization angles (top view), brief motions of short duration, or clips that mix grooming with non-grooming activities (examples are shown in Supplementary Video S2).

**Figure 10:**
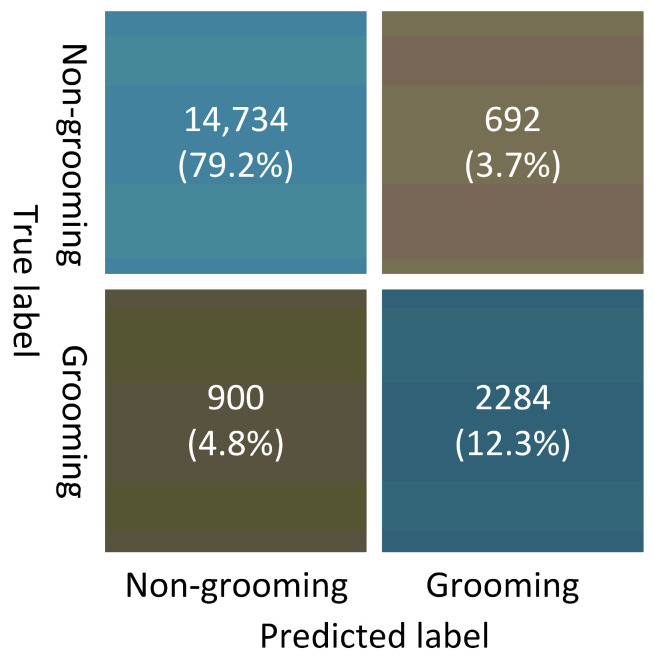
Confusion matrix of the detection results.

To obtain the confusion matrix for model performance, the dataset-dependent trained model is used to test all six datasets, including both training and validation data. The performance of the algorithm is presented in a confusion matrix showing the four standard indices (Figure 10). On average, the model achieves the following metrics: specificity of 95.5%, sensitivity of 71.7%, accuracy of 91.4%, and precision of 76.7%.

### 3.2. Grooming Activity Patterns in Pre- and Post-Drug Conditions

**Figure 11:**
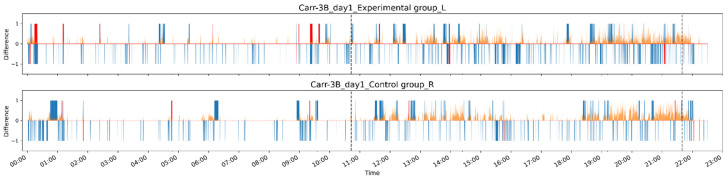
An example showing grooming activity (experimental, control). Here, correctly detected confirmed grooming clips are represented by up ward bars, and correctly detected confirmed non-grooming clips are represented by down-ward bars; blue indicates correct recognition, and red indicates incorrect recognition. The dotted line indicates day and night switching.

**Figure 12:**
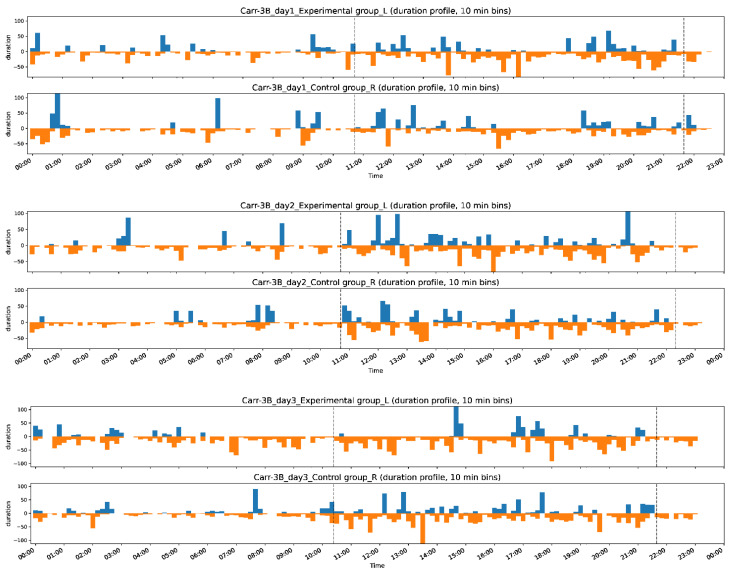
Duration time profiles over three days (blue: grooming; brown: non-grooming). The dotted line indicates day and night switching.

To minimize the ~10% error in our machine recognition of grooming behavior, we present all biological findings based on human-judged data. A general view of an exemplary grooming pattern is shown in Figure 11 on a 24 h scale (dataset Carr-3B, first day). Figure 12 shows the grooming and non-grooming activity patterns over three days, displaying the number and duration of grooming (blue bars) and non-grooming (brown bars) activities on a 24 h scale, with 10 min bins, for the same dataset as Figure 11. Similar plots of averaged grooming or non-grooming activities for each dataset are shown in Supplementary Figure S1A,B.

First, pre-drug grooming activity, in terms of both the number and duration of grooming clips, displayed a diurnal activity pattern typical of rats (i.e., more active during the night). Both experimental and control rats exhibited similar activity patterns. For post-drug grooming activity, an example of pain-related changes in grooming is shown in Figure 12 on a 24 h scale. However, it was not immediately obvious what specific pattern of grooming was altered by pain. When comparing the population data, we found no significant changes in the total grooming duration (between experimental and control rats, or between pre- and post-drug conditions, Wilcoxon signed-rank test, both *p* = 0.6) or the total number of grooming episodes (between experimental and control rats, or between pre- and post-drug conditions, Wilcoxon signed-rank test, *p* = 0.75 and *p* = 0.89, respectively) (Supplementary Figure S2A,B).

Secondly, to compare grooming duration histograms in terms of their estimated PDF profiles, we applied the two-sample Kolmogorov–Smirnov (KS) test. The *p*-values from the KS test for comparisons across rats (experimental vs. control) and within the same rat (pre-drug vs. post-drug) conditions are shown in Table 8. We found that only in the experimental group did the pre- and post-drug PDFs differ statistically, both across rats and within the same rat condition (*p* < 0.05). These results suggest that pain-induced changes in grooming duration distribution are associated with the effects of the pain inducers, rather than with the injection itself, as no significant difference was observed in the control group.

**Figure 13:**
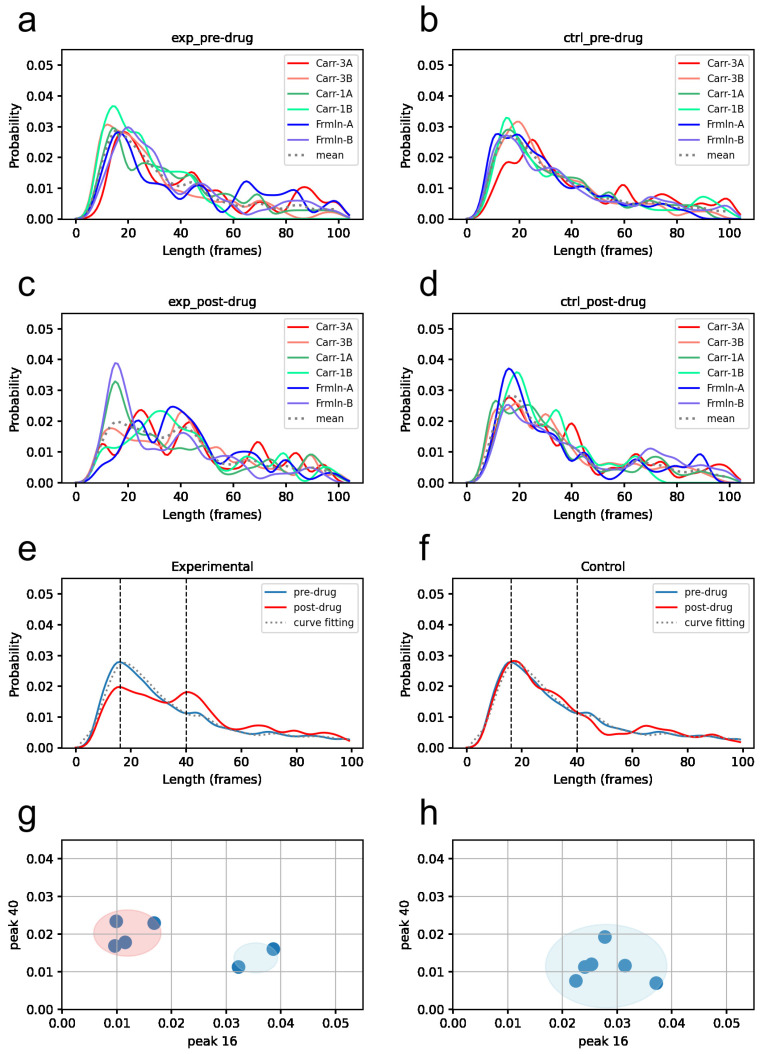
Grooming duration PDFs of all six datasets and their mean PDF. (**a**) pre-drug experimental, (**b**) pre-drug control, (**c**) post-drug experimental, (**d**) post-drug control conditions, (**e**) pre-drug condition (panels (**a**) and (**b**) averaged), post-drug condition (panel c averaged), with vertical dashed lines marking peak positions of the post-drug tracing at *x* = 16 and 40 s, (**f**) similar to (**e**) but for controls, (**g**) scatter-plot showing the *y*-intercepts of individual datasets, and (**h**) similar to (**g**) but for controls.

To further explore which parts of the grooming duration distribution are affected by pain, we carefully examined the PDFs of all six datasets, as shown in Figure 13. Figure 13a–d display the PDFs for the pre-drug experimental, pre-drug control, post-drug experimental, and post-drug control conditions. Figure 13e shows the PDFs for the pre-drug condition (averaged from panels a and b) and post-drug condition (averaged from panel c), with vertical dashed lines marking the peak positions of the post-drug trace at *x* = 16 and 40 s. Curve fitting for the pre-drug conditions across all datasets was performed using radial basis functions (details can be found in Supplementary Table S1). Figure 13f is similar to Figure 13e but for the control group. Figure 13g is a scatter plot displaying the *y*-intercepts of individual datasets (from tracings in panel a at 16 and 40 s), showing groupings (2 SD boundaries) from cluster analysis. Figure 13h is similar to Figure 13g but for the control group. For comparison, similar results from the dataset-dependent approach are shown in Supplementary Figure S3.

Based on comparisons across animals, we found that pain inducers led to the following changes in the duration distribution, most clearly observed with 3% carrageenan (datasets Carr-3A, Carr-3B): (1) a reduction in the incidence of short-duration grooming (<20 s); and (2) an increase in the incidence of longer-duration grooming (20 to 60 s), likely related to the animal licking the pain-injection site. When examining grooming episodes lasting a minute or longer (60 to 100 s), pain-related changes appeared less consistent across datasets.

While the clearest drug-induced grooming changes were observed in two datasets (Carr-3A, Carr-3B), similar changes were also seen in another two datasets: one with a lower carrageenan dose (Carr-1B) and one with short-acting formalin (Frmln-A). In contrast, the remaining two datasets (Carr-1A and Frmln-B) did not show such pain-related changes, resembling the control rats. We interpret these variable responses among animals as being due to differences in drug doses (for the same drug) and the duration of action, in addition to individual variations.

To better reflect the pain-related dual changes in grooming, we developed a response metric that captures these alterations more clearly. Specifically, we simplify the dual changes for each dataset by counting (or calculating the relative frequency, *y*-intercept) at two specific grooming durations: one at 16 s, representing short-duration grooming, and another at 40 s, representing long-duration grooming (see vertical dashed lines in Figure 13e,f). For each dataset, these two counts are plotted as *x* and *y* coordinates on a scatter plot (Figure 13g,h). We then performed cluster analysis using the K-means clustering algorithm on these data points. In Figure 13g, we identified two clusters, while in Figure 13h, we identified one cluster.

A similar analysis was conducted on the combined data points from Figure 13g,h. The results, shown in Supplementary Figure S4A, reveal two distinct clusters (with a Silhouette coefficient of 0.579, indicating moderate clustering intensity and support for non-overlapping clusters). We interpret that one of the clusters in Figure 13g (highlighted in the light blue area) overlaps with the cluster in Figure 13h. These clusters likely represent grooming behavior with no drug-induced pain. Similar clusters were also observed using the dataset-dependent approach, as shown in Supplementary Figure S4B.

**Figure 14:**
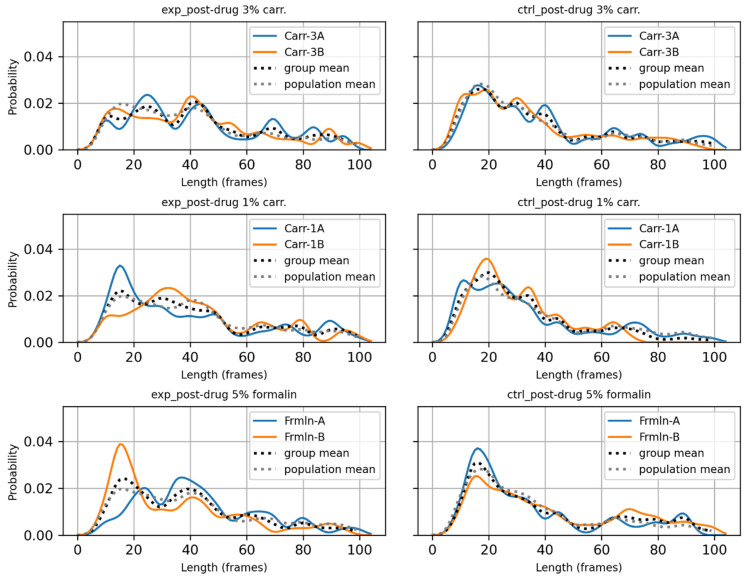
PDF profiles of the three drug groups.

The effects of different drugs and doses are visualized in Figure 14. In this figure, the post-drug PDF of each dataset within a group is shown, along with the group mean (average PDF of two rats within the same panel) and the population mean (average PDF of six rats within the same column). While the counts at 40 s appear higher for all six datasets, the counts at 16 s appear lower for four of the datasets. The corresponding pre-drug data are presented in Supplementary Figure S5.

## 4. Conclusions and Discussion

In line with our working hypothesis, two principal findings of this study are as follows: (1) the proposed two-stage activity detection method successfully detected grooming episodes at a satisfactory level (accuracy ranging from 0.873 to 0.900), offering both time and effort efficiency; and (2) pain-related activity was reflected in an increased number of grooming episodes within a specific duration range (between 20 and 60 s), particularly with longer-duration episodes (~40 s) and fewer short-duration episodes (~16 s). This pattern was most prominent in datasets where pain inducers were more effective (e.g., higher doses of carrageenan), indicating a consistent response where animals groomed for longer durations in affected areas, likely to alleviate discomfort. Conversely, datasets with lower doses or different inducers displayed more variability, suggesting that drug type and dosage influence the emergence of grooming patterns, possibly due to differences in pain intensity or behavioral responses.

In addition to this, the total number or duration of grooming remains unchanged by drug treatment, suggesting an inherent constancy in grooming behavior. Our finding of such dual-changes holds true not only for the human-judged results (which serve as the ground truth), but also for the results from dataset-dependent modeling. We propose a new response metric based on these dual-changes to indicate pain-related grooming behavior. The findings of pain-related grooming behavior, particularly with more episodes at approximately 40 s, can help direct manual efforts when necessary to review the modeled results.

The main features of our method lie in its two-stage processing. In the first stage, a significant number of grooming-unrelated episodes are filtered out. In the second stage, the MSRC network combines spatial ConvNets, temporal ConvNets, and LSTM to process the data. This model is well equipped to learn sufficient spatial and temporal information from the images, enabling it to recognize grooming activities with high accuracy. To address the poor shape of the duration histogram caused by an insufficient number of episodes, we also employ a KDE-constructed PDF for statistical comparisons.

The model’s generalization ability was evaluated using three different approaches: dataset-dependent, dataset-independent, and group-independent. With an average validation accuracy of 88.5%, this result provides strong support for its generalization capability, demonstrating its potential for accurately classifying unseen data from other rats in future experiments.

The limitations of our study are primarily biological, as outlined below. First, the sample size is relatively small, which is often a limitation in pain studies due to ethical considerations. With larger sample sizes, inter-animal variability could be reduced, and the two-cluster finding (shown in Supplementary Figure S4) could be refined and strengthened across all three modeling approaches. Second, human judgment of grooming behavior can sometimes be uncertain, particularly due to the use of single-camera images. Incorporating additional cameras could help minimize this ambiguity. Third, the observed increase in grooming episodes with a duration of approximately 40 s needs further validation, ideally by analyzing leg-licking behavior using novel algorithms, if human judgment cannot fully confirm it.

In conclusion, the proposed method is the first of its kind to offer an objective approach to pain assessment through long-term activity monitoring in animals. This method has the potential for broader applications, including assisting in the healthcare of bedridden elderly individuals in today’s increasingly robotic-driven world.

## Figures and Tables

**Table 1:** Subcutaneous injection of the six datasets, and details of video recordings.

Dataset	Day	Recorded Video Length (hh:mm:ss)	Subcutaneous Injections	
			Experimental Group	Control Group
Carr-3A	Day 1 pre-drug	22:13:05	3% carrageenan	Isoflurane gas anesthesia
	Day 2 pre-drug	23:47:23		
	Day 3 post-drug	22:16:18		
Carr-3B	Day 1 pre-drug	22:30:32	3% carrageenan	0.9% saline
	Day 2 pre-drug	23:13:20		
	Day 3 post-drug	23:02:06		
Carr-1A	Day 1 pre-drug	22:47:10	1% carrageenan	0.9% saline
	Day 2 pre-drug	23:38:38		
	Day 3 post-drug	23:21:08		
Carr-1B	Day 1 pre-drug	22:31:13	1% carrageenan	0.9% saline
	Day 2 pre-drug	22:10:55		
	Day 3 post-drug	23:42:41		
Frmln-A	Day 1 pre-drug	23:02:24	5% formalin	0.9% saline
	Day 2 pre-drug	23:02:32		
	Day 3 post-drug	22:44:34		
Frmln-B	Day 1 pre-drug	22:56:58	5% formalin	0.9% saline
	Day 2 pre-drug	23:23:43		
	Day 3 post-drug	23:08:07		

**Table 2:** Numbers of confirmed grooming clips with durations ≤100 s.

Dataset	Experimental		Control	
	Pre-Drug	Post-Drug	Pre-Drug	Post-Drug
Carr-3A	55	45	66	64
Carr-3B	85	46	81	123
Carr-1A	71	83	89	90
Carr-1B	39	59	70	54
Frmln-A	52	59	68	61
Frmln-B	67	67	71	93

**Table 3:** Details of likely grooming clips from all datasets under various conditions.

Dataset	Day	Number of Frames (2 Rats)	Experimental			Control		
			No. of Clips	No. of Frames	Length % of Video	No. of Clips	No. of Frames	Length % of Video
Carr-3A	Day 1 pre-drug	79,985	475	23,385	29.24	540	24,840	31.06
	Day 2 pre-drug	85,643	506	26,745	31.23	634	26,355	30.77
	Day 3 post-drug	80,178	519	22,630	28.22	533	22,690	28.30
Carr-3B	Day 1 pre-drug	81,032	495	16,880	20.83	428	14,120	17.43
	Day 2 pre-drug	83,600	479	16,495	19.73	491	15,050	18.00
	Day 3 post-drug	82,926	543	19,105	23.04	599	17,735	21.39
Carr-1A	Day 1 pre-drug	82,030	498	13,545	16.51	465	14,245	17.37
	Day 2 pre-drug	85,118	562	17,400	20.44	469	14,755	17.33
	Day 3 post-drug	84,068	536	17,105	20.35	496	14,325	17.04
Carr-1B	Day 1 pre-drug	81,073	523	18,210	22.46	477	16,285	20.09
	Day 2 pre-drug	79,855	458	17,165	21.50	478	16,540	20.71
	Day 3 post-drug	85,361	546	20,330	23.82	504	18,870	22.11
Frmln-A	Day 1 pre-drug	82,944	527	17,125	20.65	558	16,910	20.39
	Day 2 pre-drug	82,952	513	17,455	21.04	529	16,850	20.31
	Day 3 post-drug	81,874	466	17,005	20.77	500	17,215	21.03
Frmln-B	Day 1 pre-drug	82,618	518	16,975	20.55	498	16,440	19.90
	Day 2 pre-drug	84,223	546	16,035	19.04	518	17,530	20.81
	Day 3 post-drug	83,287	649	19,875	23.86	534	17,675	21.22
Average/day		82,709	520	18,525	22.40	514	17,690	21.40
Total		1,488,767	9359	333,465		9251	318,430	

**Table 4:** Numbers of training and validation clips.

Dataset	Training Data		Validation data		Total
	Confirmed Non-Grooming	Confirmed Grooming	Confirmed Non-Grooming	Confirmed Grooming	
Carr-3A	2112	453	528	114	3207
Carr-3B	1947	481	487	120	3035
Carr-1A	1964	456	492	114	3026
Carr-1B	2019	369	505	93	2986
Frmln-A	2101	373	526	93	3093
Frmln-B	2196	414	549	104	3263
Total	12,339	2546	3087	638	18,610

**Table 5:** Comparison with other behavior recognition methods.

Methods	ConvNet	Training Accuracy	Validation Accuracy
TSN [22]	BN-Inception	0.794	0.762
TS-LSTM [25]	ResNet-101	0.857	0.813
Ours	ResNet-50	0.911	0.900

**Table 6:** Results of the dataset-independent approach.

Training Data	Validation Data	Training Accuracy	Validation Accuracy
Carr-1B, Carr-3A, Carr-3B, Frmln-A, Frmln-B	Carr-1A	0.907	0.903
Carr-1A, Carr-3A, Carr-3B, Frmln-A, Frmln-B	Carr-1B	0.908	0.886
Carr-1A, Carr-1B, Carr-3B, Frmln-A, Frmln-B	Carr-3A	0.914	0.839
Carr-1A, Carr-1B, Carr-3A, Frmln-A, Frmln-B	Carr-3B	0.909	0.884
Carr-1A, Carr-1B, Carr-3A, Carr-3B, Frmln-B	Frmln-A	0.909	0.882
Carr-1A, Carr-1B, Carr-3A, Carr-3B, Frmln-A	Frmln-B	0.906	0.896
Average		0.909	0.882

**Table 7:** Results of the group-independent approach.

Training Data	Validation Data	Training Accuracy	Validation Accuracy
1% carrageenan group + 5% formalin group	3% carrageenan group	0.915	0.872
3% carrageenan group + 5% formalin group	1% carrageenan group	0.905	0.882
1% carrageenan group + 3% carrageenan group	5% formalin group	0.906	0.863
Average		0.909	0.873

**Table 8:** Results of KS test showing *p* values for across and same rat comparisons. Note: ‘across rats’ compares across experimental and control rats in the same dataset; ‘same rat’ compares the same rat between pre- and post- drug conditions. (* indicates *p*-value < 0.05).

Dataset	Across Rats		Same Rat	
	Pre-Drug	Post-Drug	Experimental	Control
Carr-3A	0.436	0.009 *	0.014 *	0.053
Carr-3B	0.533	0 *	0 *	0.751
Carr-1A	0.773	0.027 *	0.038 *	0.430
Carr-1B	0.234	0 *	0 *	0.321
Frmln-A	0.184	0 *	0 *	0.162
Frmln-B	0.377	0 *	0.025 *	0.068
Mean	0.086	0 *	0 *	0.074

**Figure A1:**
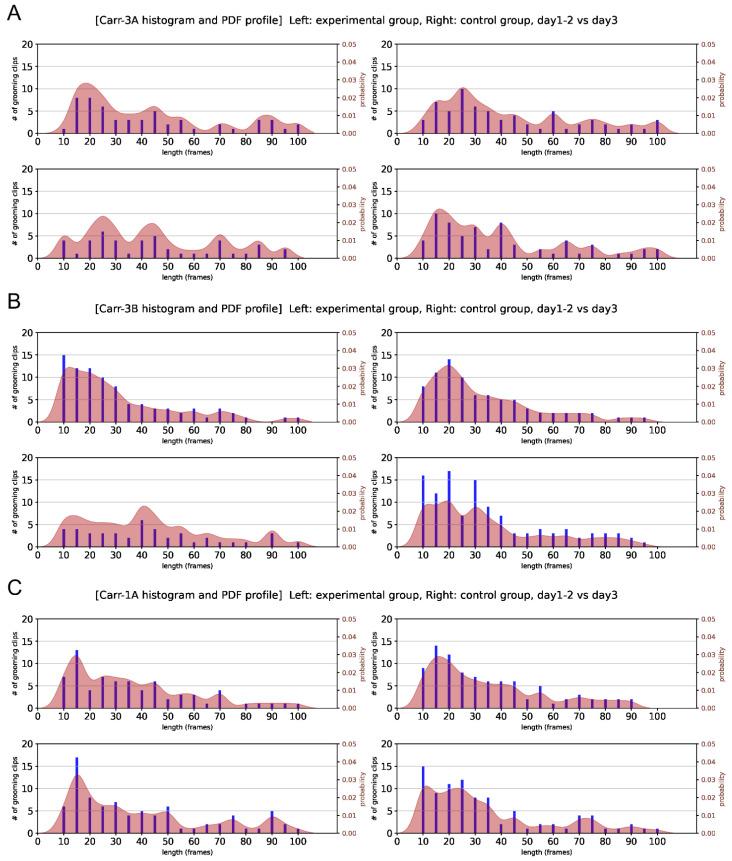

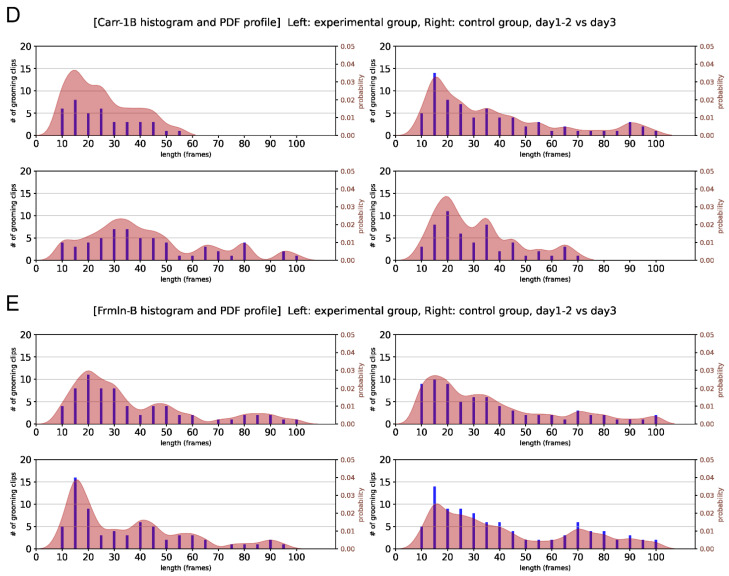
Grooming duration histograms overlaid with the corresponding reconstructed PDFs. Captions similar to Figure 5. Histograms and PDF profiles of (**A**) Dataset Carr-3A, (**B**) Dataset Carr-3B, (**C**) Dataset Carr-1A, (**D**) Dataset Carr-1B, and (**E**) Dataset Frmln-B.

## Data Availability

The data presented in this study are available on request from the corresponding author due to restrictions imposed by hospital regulations and confidentiality agreements.

## Supplementary Materials

The following supporting information can be downloaded at: https://www.mdpi.com/article/10.3390/bioengineering11121180/s1, Figure S1: Time profiles over three days; Figure S2: Comparison of the pre-drug and post-drug conditions in the experimental and control rats; Figure S3: Grooming duration PDFs of all six datasets and their mean PDF; Figure S4: Cluster analysis of the combined data in Figure 13g,h; Figure S5: PDF profiles in the pre-drug condition of all six groups; Table S1: Curve fitting of the average pre-drug grooming duration PDF; Video S1: Examples of human-confirmed grooming behaviors; Video S2: Examples of falsely identified grooming clips.
