# Supplementary material for: Detection of Rat Pain-Related Grooming Behaviors Using Multistream Recurrent Convolutional Networks on Day-Long Video Recordings
Source: Bioengineering (Basel). 2024 Nov 21;11(12):1180. doi: 10.3390/bioengineering11121180 (PMC11673758; doi:10.3390/bioengineering11121180)
Supplement: Supplementary file 1 [file bioengineering-11-01180-s001.zip › supplementary_resource.pdf]

Supplementary Information

Fig. S1 Time profiles over three days. (A) showing similar pictures of duration with mean and SD from all six datasets. (B) showing counts instead of 'duration' of grooming and non-grooming episodes of the population (mean, SD of six datasets).

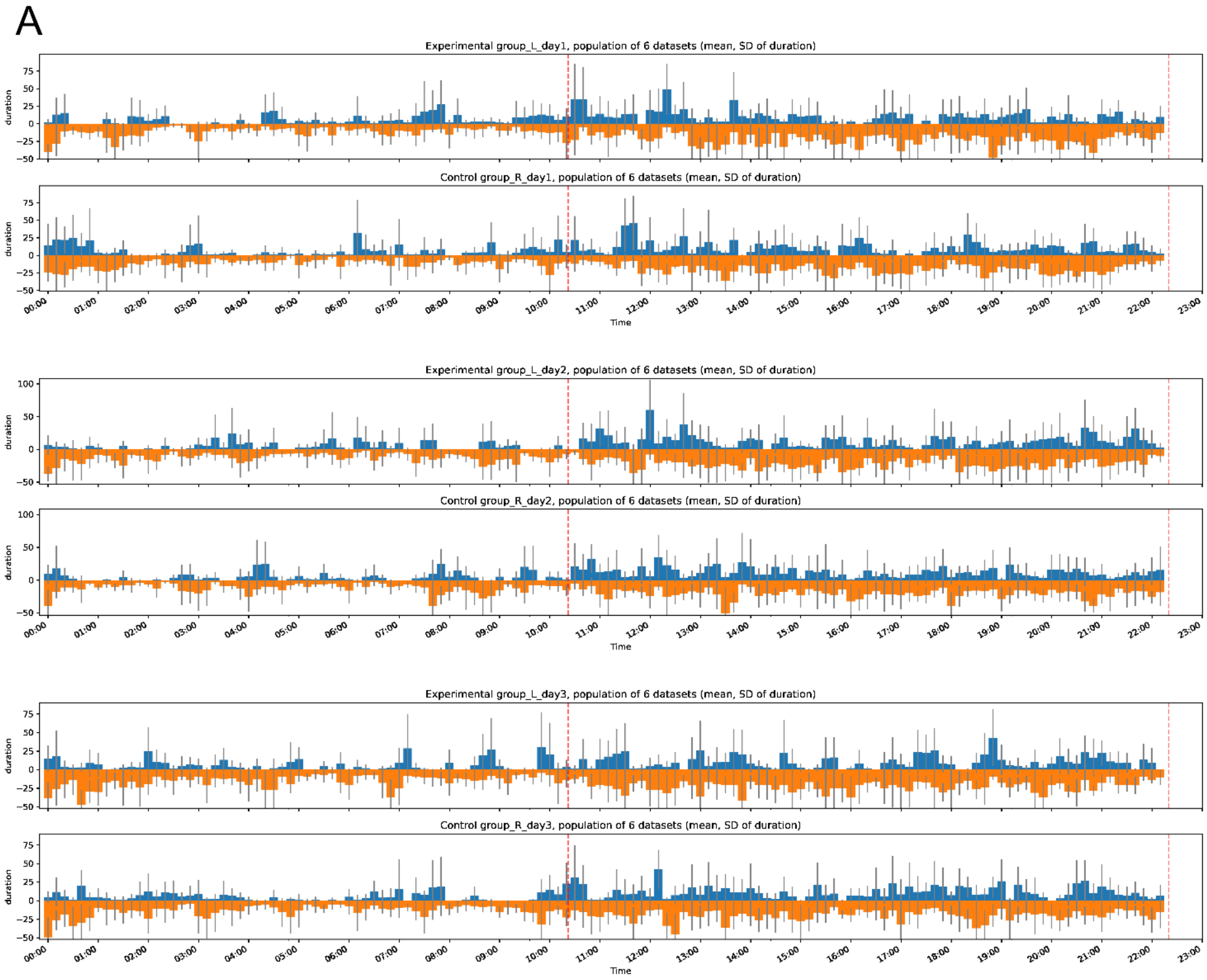

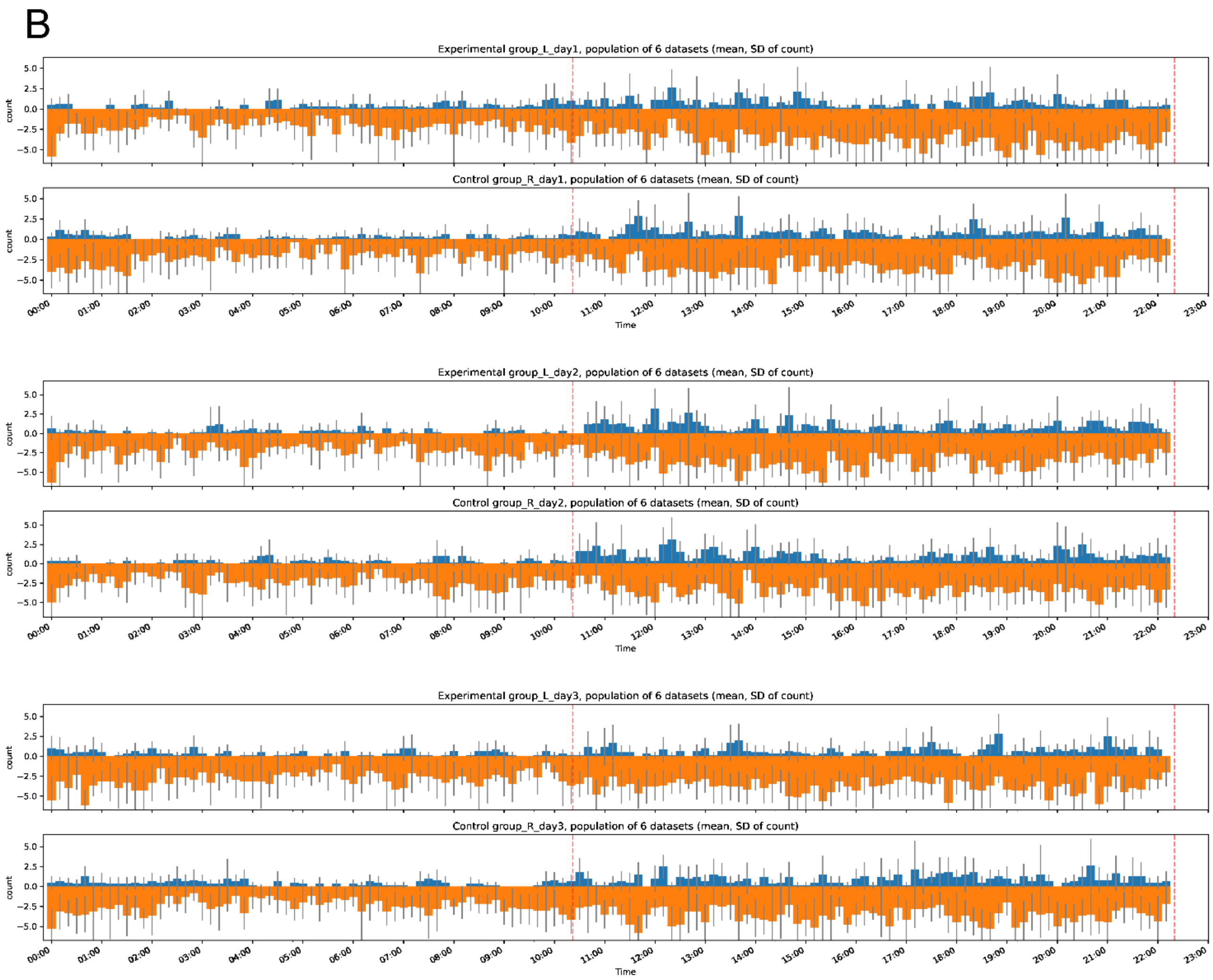
B
Experimental group_L_day1, population of 6 datasets (mean, SD of count)
Control group_R_day1, population of 6 datasets (mean, SD of count)
Experimental group_L_day2, population of 6 datasets (mean, SD of count)
Control group_R_day2, population of 6 datasets (mean, SD of count)
Experimental group_L_day3, population of 6 datasets (mean, SD of count)
Control group_R_day3, population of 6 datasets (mean, SD of count)
count
Time

Fig. S2 Comparison of the pre-drug and post-drug conditions in the experimental and control rats. (A) Total grooming durations shown for the pre-drug and post-drug conditions in the experimental and control rats. Wilcoxon signed-rank test showed no difference between pre- and post- drug conditions (p=0.6), nor between experimental and control groups (p=0.6). **(B)** Total number of grooming episodes shown for the pre-drug and post-drug conditions in experimental and control rats. Wilcoxon signed-rank test again showed no difference between pre- and post- drug conditions (p=0.89), nor between experimental and control groups (p=0.75).

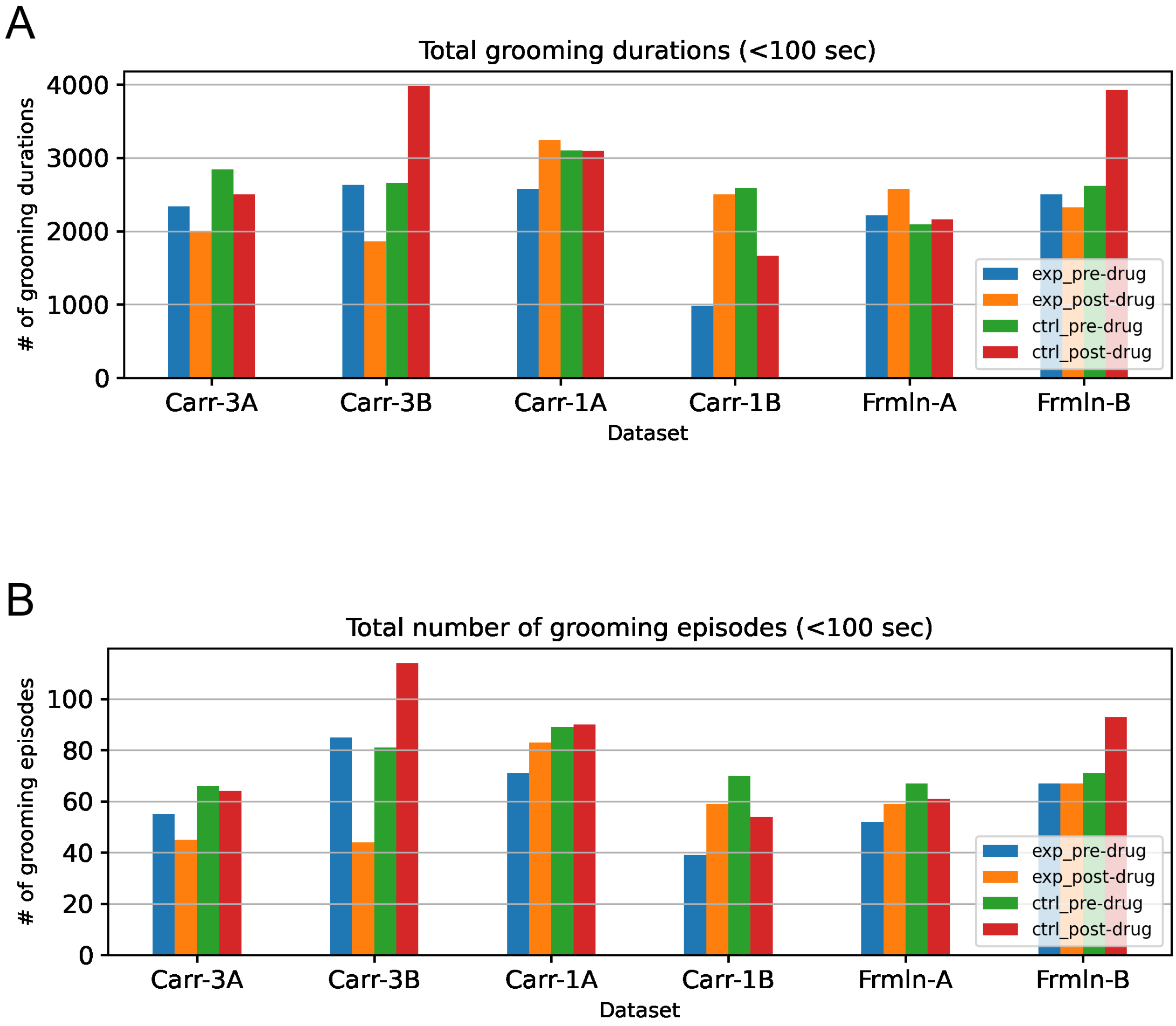

Fig. S3 Grooming duration PDFs of all six datasets and their mean PDF (same as **Fig 13)** except on model results from dataset-dependent approach, instead of from human-judgment.

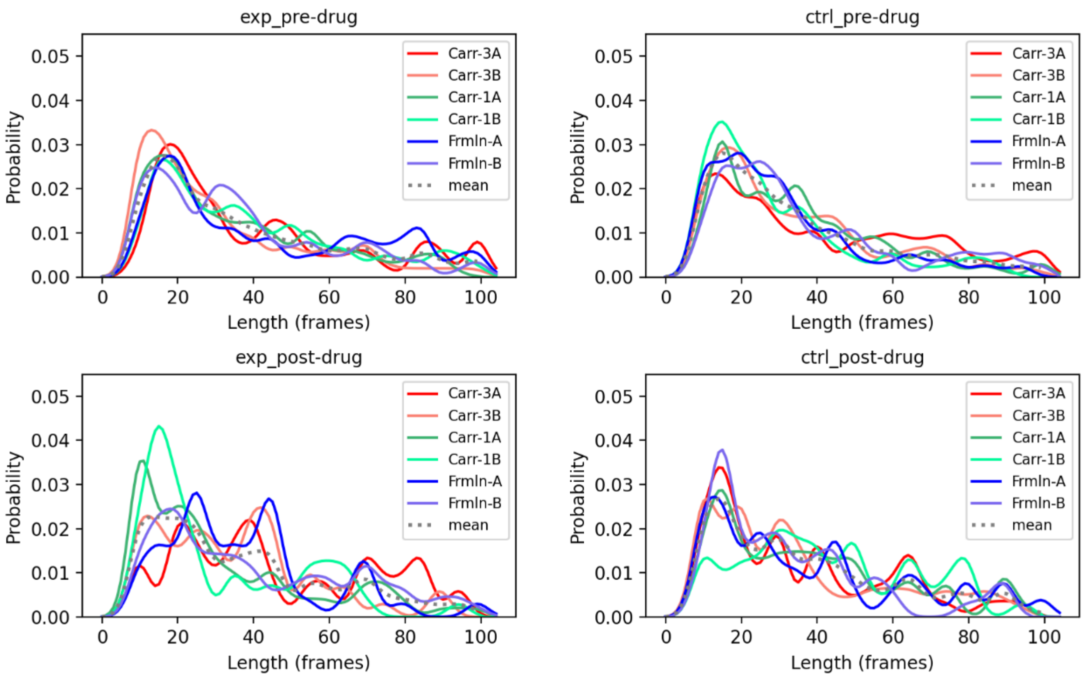

Fig. S4 Cluster analysis of the combined data in Figure 13g,h, showing center and 3 SD boundaries for two clusters **(A)**. Here, the Silhouette coefficient is 0.579. For comparison, similar results from the dataset-dependent approach are shown in **(B)**. Here, the Silhouette coefficient is 0.501.

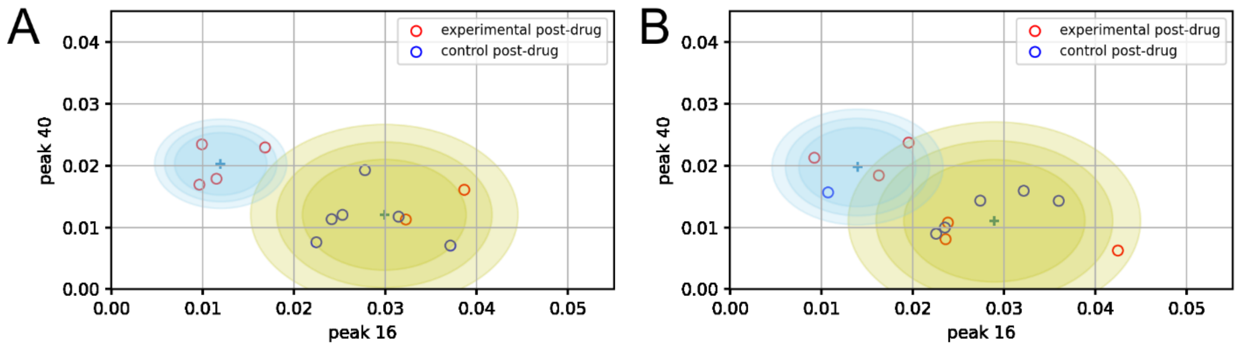

Fig. S5 PDF profiles in the pre-drug condition of all six groups (different pain inducer protocols). Captions similar to **Fig 14**. Note similar shapes across groups.

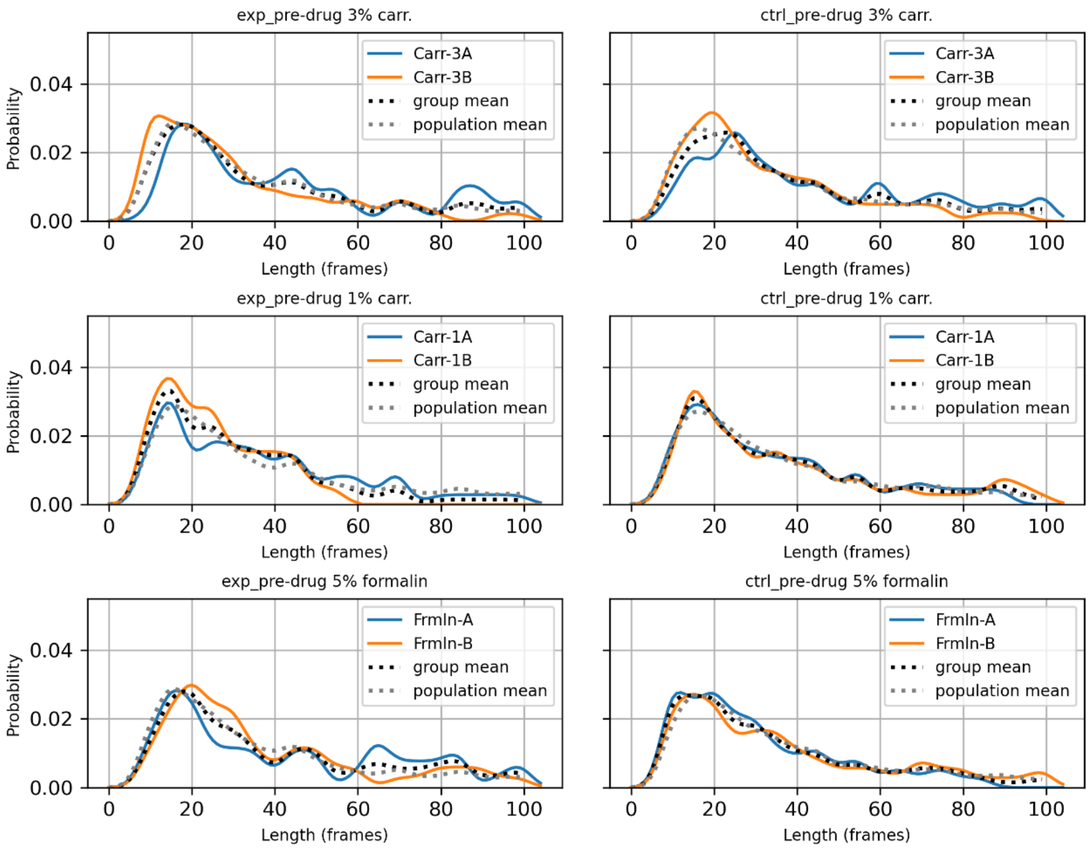

Table S1 Curve fitting of the average pre-drug grooming duration PDF, based on five radial basis functions (MSE =1.025×10^{-6}, R^2 =0.983). Parameters of the fitted model are:

k	Weight (*w*)	Center (*c*)	Bandwidth (σ)
1	-0.096	41.2	-582.5
2	-0.021	-1.5	-9.7
3	0.047	31.7	-344.5
4	0.052	100.4	-3,458.8
5	0.017	10.5	-37.8
